# mTOR Regulation of Metabolism in Hematologic Malignancies

**DOI:** 10.3390/cells9020404

**Published:** 2020-02-11

**Authors:** Simone Mirabilii, Maria Rosaria Ricciardi, Agostino Tafuri

**Affiliations:** 1 Department of Clinical and Molecular Medicine, Sapienza University of Rome, 00185 Rome, Italy; simone.mirabilii@uniroma1.it (S.M.); mariarosaria.ricciardi@uniroma1.it (M.R.R.); 2 Hematology, “Sant’ Andrea” University Hospital, Sapienza University of Rome, 00185 Rome, Italy

**Keywords:** mTOR, hematologic malignancies, cell metabolism

## Abstract

Neoplastic cells rewire their metabolism, acquiring a selective advantage over normal cells and a protection from therapeutic agents. The mammalian Target of Rapamycin (mTOR) is a serine/threonine kinase involved in a variety of cellular activities, including the control of metabolic processes. mTOR is hyperactivated in a large number of tumor types, and among them, in many hematologic malignancies. In this article, we summarized the evidence from the literature that describes a central role for mTOR in the acquisition of new metabolic phenotypes for different hematologic malignancies, in concert with other metabolic modulators (AMPK, HIF1α) and microenvironmental stimuli, and shows how these features can be targeted for therapeutic purposes.

## 1. mTOR Structure and Function

The mammalian Target of Rapamycin (mTOR) is a kinase involved in the PI3k/PTEN/Akt axis, which plays a key role in the control of many biological processes, including cell growth and survival, protein translation, ribosomal biogenesis, autophagy, and metabolism [1,2,3].

Originally identified in the yeast *Saccharomyces cerevisiae*, mTOR is a pleiotropic serine/threonine kinase of 289kDa, which shows a terminal COOH catalytic domain with a high sequence homology with PI3K [4].

mTOR is composed of 2549 amino acids and contains up to 20 tandem repeated HEAT motifs, a repeated structural motif composed of two tandem anti-parallel α-helices linked by a short loop, which work as a scaffold for a protein-protein interaction [5].

It operates within two multiprotein complexes, mTORC1 and mTORC2, which phosphorylate a different set of substrates coordinating different physiological cell functions. mTORC1 includes mTOR (the catalytic subunit of the complex), the regulatory-associated protein of mTOR (Raptor), the DEP domain-containing mTOR-interacting protein (Deptor), the mammalian lethal with SEC13 protein 8 (mLST8), the raptor binding protein PRAS40 and the FK506-binding protein 38 (FKBP38). mTORC2 is conversely composed of mTOR itself, the rapamycin-insensitive companion of mTOR (Rictor), mLST8, the mammalian stress-activated map kinase-interacting protein 1 (mSIN1), a protein observed with Rictor (Protor-1) and Deptor [3,6].

The two complexes display different response to rapamycin and its derivatives (rapalogs), being mTORC1 sensitive to the inhibitory effects of these immunosuppressant, while mTORC2 proved insensitive. However, in some cell types, it has been shown that prolonged treatment with rapamycin and rapalogs can indirectly inhibit the formation and activity of the TORC2 complex [7].

Various upstream events can lead to the activation of mTORC1, mostly convergent on Akt. For instance, Akt can inactivate through phosphorylation either TSC2 (tuberous sclerosis protein 2) in the TSC1–TSC2 complex, which negatively regulates mTORC1, or PRAS40, antagonizing its activation by Rheb, respectively [8,9].

In response to nutrient availability and growth factors, activated mTORC1 regulates protein translation by phosphorylating p70S6 (p70S6K) and 4E-BP1 kinases, which in turn phosphorylate the S6 protein kinases (p70S6K1/2) and the eukaryotic initiation factor 4E (eIF4E)-binding proteins (4E-BP1/2), which are involved in the translation process [6,10]. In particular, the phosphorylated S6K enhances the translation of mRNAs that have 5′ polypyrimidine rich sequences [11,12]. Conversely, phosphorylation of 4E-BP1 causes it to release eIF4E, which binds the mRNA 5′-cap, thus allowing the translation to begin [13].

**Figure 1:**
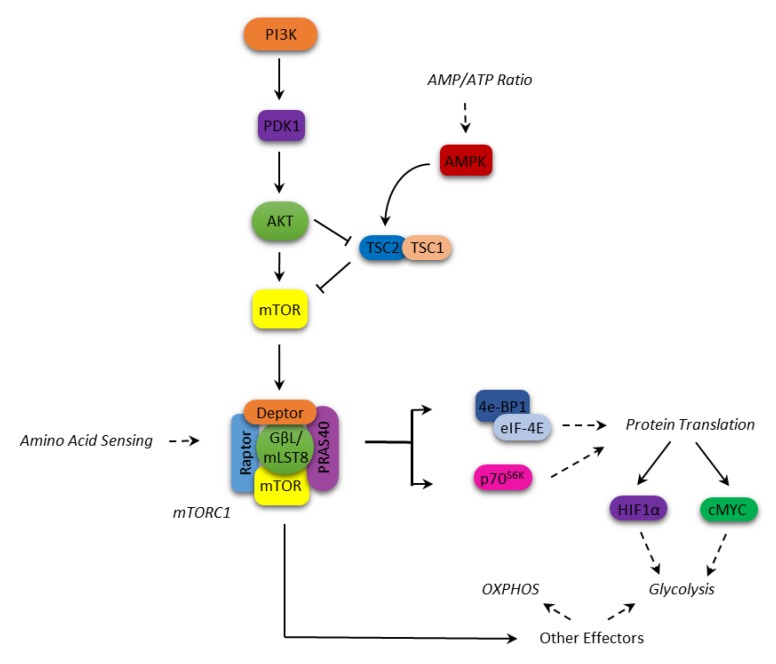
mammalian Target of Rapamycin (mTOR) signaling and cellular metabolism.

In addition, the mTORC1 complex regulates the expression of key proteins such as cyclin D1, STAT3, Bcl-2, Bcl-xL, Mcl-1, thus promoting cell proliferation and survival [14,15,16]. As for the metabolic function, mTORC1 is a central signaling node in coordinating the metabolic cell response (Figure 1). mTORC1 is involved in metabolic reprogramming by increasing glycolysis and macromolecules biosynthesis through transcriptional, translational, and post-translational mechanisms mediated by its substrates, p70S6K and 4E-BP [17,18,19]. Among these mechanisms, mTOR enhances the translation of critical metabolic mediators such as c-Myc and hypoxia-inducible factor 1 alpha (HIF1α) [20]. c-Myc upregulates many genes involved in the glycolytic process such as glucose transporters, hexokinase 2 (HK2), phosphofructokinase (PFKM), and enolase 1 (ENO1) [21]. HIF1α is an oxygen-sensing molecule that is stabilized in hypoxic condition, and translocates to the nucleus initiating the transcription of hypoxic response genes [22]. Its action on cell metabolism includes an increased glucose uptake, a higher glycolytic flux and a lower oxidative phosphorylation (OXPHOS) [23]. On the other hand, AMP-activated protein kinase (AMPK) acts as an mTOR inhibitor; it is a serine/threonine kinase that is able to respond to the fluctuating intracellular AMP levels, shutting down energy-depleting processes in favor of catabolic pathways, such as fatty acid oxidation and autophagy, when the AMP level rises [24]. Once activated, AMPK inhibits mTOR through the activation of TSC2 [24].

However, it was also reported that mTORC1 could promote anabolic metabolism independently from p70S6K and 4E-BP1 [25]. The authors demonstrated that mTOR regulates oxygen consumption and oxidative capacity independently from these effectors. Energy/nutrition depletion and stress signals seem indeed indirectly sensed by mTORC1 via the LKB1-AMPK cascade [26].

mTORC activity and, above all, its regulation mechanisms are less well known. While mTORC1 is mostly involved in sustaining cell growth, proliferation, and survival by controlling the translation machinery, autophagy or mitochondrial biogenesis, the main function attributed to mTORC2 represents the regulation of the actin cytoskeleton polarity mediated by the Rho1/Pkc1/MAPK cell cascade [27,28].

The assembly of the actin cytoskeleton in response to mitogenic signals is promoted by mTORC2 through the activation of several cytoskeletal regulators, such as TCP-1, ROM2 and Ypk [29,30,31,32]. It was observed that the deregulation of mTORC2 activity causes the alteration of cytoskeletal actin [27] and impacts cell motility in some types of tumor cells [33].

It has been widely reported that nutrient availability and cellular energy status do not seem to be necessary for the activation of mTORC2 [34,35,36]. In contrast, in proliferating cells, the activation of mTORC2 requires the close interaction between the protein multicomplex and the ribosome, underlining a reciprocal interaction between the two complexes [34]. Indeed, since mTORC1 regulates ribosome biogenesis, which is crucial to determine cell growth capacity, it indirectly controls mTORC2 activity [34]. In turn, mTORC2 promotes the Akt-TSC1/TSC2-mediated mTORC1 activity [37]. Connected to the PI3K/AKT signaling pathway, it is described that mTORC2 phosphorylates Akt on Ser473. However, a crosstalk between mTORC1 and mTORC2 and with other metabolic pathways has been reported [38].

## 2. Cancer Metabolism and Cell Signaling

We have learned from the literature that alterations in cellular metabolism pathways are manifest in cancer cells as compared to normal tissue cells [39,40,41].

Nuclear and mitochondrial alterations in the genome of cancer cells, pressing for an increased import of bioenergetic substrates and/or an increased generation of biosynthetic intermediates needed for cell growth and proliferation, are tightly linked to the altered cancer metabolism. On the other hand, the products of metabolism, especially ROS, damaging cells can promote oncogenic DNA mutations [42,43].

Among the altered metabolic pathways of cancer cells, the increased glycolytic flux, the exploitation of alternative carbon source like glutamine, and the increase in fatty acid metabolism are recognized [39,44]. The alteration of the metabolic processes, however, differs from tumor to tumor, because of the close interaction between the cells and the tumor microenvironment, where the concentrations of nutrients such as glucose and glutamine, or gas such as oxygen, are spatially and temporally heterogeneous [45,46].

Moreover, there are differences, in terms of energy requirements and biomass production, between the differentiated and undifferentiated cells, as well as in the metabolic needs and the related regulatory mechanisms between the proliferating and quiescent cells [40,44,47]. This concept needs to be taken into account when approaching metabolic analysis in different leukemia settings.

Recently, several authors have shown that many of the Signal Transduction Pathways (STPs) aberrantly activated in cancer cells actually converge on the deregulation of common metabolic mechanisms responsible for cell growth and survival [40,48,49]. The instructional metabolic reprogramming from signaling is critical for cellular homeostasis and cell fate. However, the relationship between altered cellular signaling and reprogrammed metabolism is not unidirectional and several feedback mechanisms, in which metabolites can control signal fluxes through specific sensor kinases that monitor the cell bioenergetic status are found to be active in cancer cells [50,51].

In this scenario, mTOR, as an orchestrator of nutrient sensor, signaling processor, and cell growth regulator, represents one of the main actors in coordinating cell growth, division, and survival with cell metabolic activity [3].

## 3. mTOR and the Metabolism of Hematologic Malignancies

### 3.1. Acute Myeloid Leukemia

Different studies ascribe an overall glycolytic metabolism to acute myeloid leukemia (AML) cells, with a conspicuous lactate production and an active tricarboxylic acid (TCA) cycle, in order to fulfil biosynthetic purposes. In a metabolomic study on over 400 patients, Chen et al. detected a higher glycolytic flux in AML samples when compared to healthy controls, along with a compensatory pyruvate generation from amino acids in order to feed the TCA cycle [52]. Increased glycolytic rates were already reported by Herst et al., although with a variable extent, dividing patients into two subgroups based on the glycolysis rate [53]. Interestingly, both groups confer to glycolysis a prognostic meaning, encouraging further studies to explore this feature. To confirm the importance of glycolysis in AML cells, a thorough work by Wang et al. demonstrated, through the use of murine models and a serial transplantation approach, that the conditional deletion of the two last enzymes of aerobic glycolysis, pyruvate kinase and lactate dehydrogenase, profoundly affect the viability of leukemic-like cells [54]. As mentioned, AML cells can utilize carbon sources other than glucose. One such sources is glutamine, as it has been long known that this amino acid is essential to AML cells [55]. Recently, fatty acid utilization has come into attention: our group, along with others, has demonstrated that the utilization of these compounds plays a major role in AML bioenergetics balance [56,57,58].

In this bioenergetic picture, the glycolytic phenotype is at least partially caused by mTOR hyperactivation, as it is described as a controller of the glycolytic process in several studies that were carried out on AML cells. Liu et al. observed that mTOR inhibition through rapamycin caused a decrease in glucose uptake on AML cell lines [59]. A similar experience has been reported by our group, as the inhibition of the PI3K/mTOR axis signaling caused a drop in the glycolytic fluxes, along with a reduction in OXPHOS [60]. Additionally, Poulain et al. directly correlated the extent of glycolytic flux with the activation status of mTORC1: analysis by gene expression profile of AML cell lines treated with rapamycin revealed a downregulation of genes involved in the glycolytic pathway, while from the metabolic perspective rapamycin caused a reduction of glycolytic flux with a concomitant shift on OXPHOS, as testified by the increase of TCA cycle enzymes [61]. Coherently, rapamycin protected AML cell lines from apoptosis induced by either glucose deprivation or 2-deoxy-glucose (2DG) glycolysis inhibition [61]. Much uncertainty remains on the reverse mechanism, i.e., what happens to mTOR when glycolysis is inhibited. Pradelli et al. reported the mTOR inactivation in AML cells exposed to 2DG, due to the action of AMPK [62]. To further complicate the picture, Estañ et al. observed instead that 2DG caused AMPK inhibition and a concomitant activation of the Akt/mTOR axis [63]. This opposite mechanism of action might be partially explained by the different cell models used by these studies (U937 in the first; HL60, NB4, and THP-1 in the second) that can reflect the heterogeneity in genetic and clinical features of the AML. Indeed, Pereira et al. confirmed this heterogeneity by measuring glycolysis of AML cell lines in correlation with Akt/mTOR and AMPK activation status, finding a fluctuation in the glycolytic flux across the different cell lines, correlated with a different intracellular signaling [64]. Of interest, in the KG1 cell line they found a simultaneous activation of AMPK and mTOR, subverting the canonical view that dictates an antithetic role for these two kinases [64].

Recent evidence has identified PFKFB3 (6-phosphofructo-2-kinase/fructose-2,6-biphosphatase 3) as a probable target of the mTOR signaling directly involved in the glycolytic process: Feng and Wu, in fact, observed in their work that mTOR interacts with this enzyme upregulating aerobic glycolysis [65]. Exposure to rapamycin reverts this glycolytic metabolism, downregulating PFKFB3 protein levels [65].

Previous data demonstrated that this glycolytic phenotype, mediated by mTOR, is somehow exacerbated by extracellular stimuli originating from the microenvironment. In fact, the contact with the stromal compartment appears to increase the glycolytic flux of AML cells, through a mechanism involving a chemokine, CXCL12, recognized by its receptor CXCR4, which in turn activates mTOR [66]. This observation may constitute one of the factors involved in the chemoresistance, as it is widely accepted that the microenvironment protects leukemic cells from therapeutic agents [67]. Since glycolysis is another factor associated with resistance [68], the emergence of refractoriness may be also explained by the stroma-mediated upregulation of glycolysis.

In addition to the glycolytic process, mTOR has been considered as central in the regulation of amino acid homeostasis and usually becomes inactive in case of amino acid deficiency, leading to autophagy and decreased protein biosynthesis [69]. This mechanism seems to be confirmed also in AML cells, as mTOR inactivation in case of either glutamine depletion or L-asparaginases-mediated degradation leads to the autophagic process activation in an attempt by the cells to scavenge the building blocks for survival [70].

Of interest, a peculiar metabolism seems to be associated with the resistance to PI3K/mTOR axis inhibitors: a metabolomic study carried out on 30 primary samples exposed to 4 molecular inhibitors (rapamycin, GDC-0941, human insulin, indomethacin), targeting this pathway with different selectivity, identified the proline/glutamine and the arachidonic acid metabolism as markers of AML cell resistance to these agents [71]. Table 1 summarizes the effect of mTOR and metabolic enzyme inhibitors in AML.

### 3.2. Chronic Myeloid Leukemia

Earlier reports observed an upregulation of glycolysis in chronic myeloid leukemia (CML) cells, promoting the idea that the metabolism of these cells can be characterized by the Warburg effect, the glucose fermentation to lactate even in the presence of oxygen. Two independent studies detected a glycolytic phenotype in CML cells, with a reduction of the glycolytic flux following the exposure to the Bcr-Abl inhibitor Imatinib in those cells that proved sensible to its action [72,73]. This reduction has been recently confirmed by De Rosa et al., who observed the downregulation of hexokinase II and lactate dehydrogenase (two enzymes involved in the aerobic glycolysis) in CML cell models [74]. In parallel, Baldwin’s group detected an upregulation of the glucose transporter GLUT-1 and a consequent increase in glucose import in hematopoietic cell lines transfected with Bcr-Abl [75]. More recently, Sontakke et al. confirmed the aerobic glycolysis and the upregulation of glucose transporters as SLC2A1/3 in normal cord blood progenitors engineered for Bcr-Abl expression [76]. Interestingly, they found a concomitant upregulation of glutaminolysis, probably to keep the TCA cycle active despite the pyruvate subtraction [76]. More generally, the increased glycolytic flux has been associated with the resistance to tyrosine kinase inhibitors [77,78]. A different picture is emerging regarding the metabolism of CML stem cells, as multiple works stated the relevance of an oxidative phenotype in this cell population. From a transcriptomic perspective, these cells seem to upregulate genes involved in the OXPHOS when compared to CD34+ cells from healthy donors. Authors found a peculiar pattern of expression, with an upregulation of mitochondrial respiratory chain complex 1 and 2, and a downregulation of complex 3, giving rise to a defective OXPHOS and a consequent production of ROS [79]. Accordingly, two recent reports focusing on CML stem cell metabolism confirmed this oxidative phenotype, fueled by fatty acid oxidation [80], and driven by a SIRT1/PGC1-α signaling axis [81]. Importantly, targeting this metabolism, either with a specific agent (tigecycline) or a SIRT1 knockout led to the impairment of CML stem cell functions, showing synergistic interaction with tyrosine kinase inhibitors [80,81]. 

These observations seem to suggest a reprogramming of CML cell metabolism, starting from an oxidative phenotype in CML stem cells, then shifting toward a more glycolytic one in later stages of the disease.

Relatively few reports have focused on mTOR activity on metabolism in the CML setting.

High levels of ROS, caused by a sustained mitochondrial activity, have been linked to the PI3K/Akt/mTOR axis activation by Bcr-Abl signaling. This mitochondrial activity was fueled mainly by glucose, as the exposure of CML cells to 2DG lowered the ROS levels [82]. Similarly, the inhibition of this axis with a PI3K (wortmannin) or an mTOR (rapamycin) inhibitor caused a reduction of ROS, thus reaffirming the action of this signaling module on the glycolytic metabolism [82]. The control over glucose fate by Bcr-Abl/mTOR seems to rely on the activation of S6K1 [83]. Interestingly, the inactivation of S6K1 and the consequent impairment of glycolysis did not induce apoptosis, but caused a metabolic shift to fatty acid oxidation [83]. Shinohara et al. reported that mTOR mediates the expression regulation by Bcr-Abl of the balance between the two pyruvate kinase (PKM) isoforms 1 and 2 [84]; this glycolytic kinase is crucial in the control of glucose fate, between aerobic glycolysis and OXPHOS [85]. Finally, a sustained activity of the PI3K/Akt/mTOR signaling module has been reported to participate in the glycolytic phenotype of adriamycin-resistant CML cells [86].

### 3.3. Acute Lymphoblastic Leukemia

Similar to AML and CML, acute lymphoblastic leukemia (ALL) cell metabolism seems to be driven by the aerobic glycolysis, at least in the B lineage: when compared to normal cells, ALL cells show higher expression levels of glucose transporters (GLUT-1), an increased lactate production and a vulnerability to glycolysis inhibition [87]. The presence of fusion genes, like BCR-ABL, seems to further enhance this kind of metabolism [88]. Moreover, higher glycolytic rates are involved in resistance to chemotherapeutic agents, like daunorubicin [89]. The mitochondrial energy machinery seems however to be intact, as these cells are able to shift from glycolysis to OXPHOS fueled by autophagy under stress condition like exposure to glucocorticoids [90]. T cell ALL, conversely, appear to be less glycolytic and more oxidative [91]. NOTCH-1, a transmembrane receptor commonly activated in this leukemia subtype [92], appears to be implicated in this metabolic phenotype, driving these cells towards the use of glutamine to feed the TCA cycle [93].

In B-ALL, this glycolytic phenotype emerges from the interplay between mTOR, HIF1α and the hypoxic microenvironment, as showed by Frolova et al. [94]. They demonstrated, through ALL blasts and stromal cell co-culture, that the contact with microenvironment stimulates, under hypoxic condition, a signaling through the PI3K/Akt/mTOR axis, along with MAPK activation, stabilizing HIF1α and inducing the glycolytic phenotype [94]. The inhibition of mTOR with everolimus reverted this glycolytic phenotype, downregulating hexokinase II expression and reducing lactate generation [94]. Additionally, it has been reported that mTOR reacts during metabolic stress, such as 2DG exposure, cooperating with AMPK to lower Mcl-1 expression, especially in Bcr-Abl positive ALL, a mechanism that can potentiate the effect of TKI inhibitors in this setting [95]. Beside glycolysis, mTOR seems to participate in the metabolism control of important cofactors, such as thiamine, which is required for a large number of enzymes to be functionally active. Targeting this molecule with specific thiaminases causes a series of metabolic repercussions, such as a decrease of mitochondrial respiration and an increase of glycolysis, which can be reverted by the effect of rapamycin [96]. This constitutes an indirect proof of the role of mTOR in thiamine metabolism, which prompts further investigation in the leukemic setting.

In T-ALL, Kishton et al. depicted a complex picture in which mTOR is under the strict control of AMPK: microenvironmental stimuli activates Notch, which signals through mTOR for the aerobic glycolysis [91]. This metabolism, however, cannot be sustained by those cells, causing a shortage of ATP, which activates AMPK [90]. The latter then inactivates mTOR, causing a shift to a more oxidative metabolism [91]. In this context, mTOR seems therefore to drive cells toward a more sustained metabolism, with higher fluxes of glycolysis followed by an OXPHOS upregulation. An indirect evidence has in fact been reported by Fernández-Ramos et al., who observed that 6-mercaptopurine inhibits mTOR through AMPK activation, consequently reducing glucose and glutamine consumption by T leukemia cells [97]. In Table 2, the metabolic effects of mTOR inhibition in B- and T-ALL are reported.

### 3.4. Chronic Lymphocytic Leukemia

The metabolic features of chronic lymphocytic leukemia (CLL) are perhaps the best characterized among the hematological malignancies, as there is a general consensus to place these cells in the oxidative metabolic phenotype, without a clear manifestation of the Warbug effect [98]. Additionally, both glutamine and fatty acids concur to fuel the TCA cycle activity [99], conferring to CLL cells a metabolic plasticity that allows them to survive in the different body districts [100]. The rate of OXPHOS has been correlated with the degree of the disease aggressiveness: Gandhi’s group observed that CLL cells show variable respiration rates, and cells with higher rates were from patients characterized by unfavorable prognostic markers, such as a higher Rai score, beta 2 microglobulin (β2M), Zap70, and unmutated immunoglobulin heavy chain (IGHV) encoding genes [101]. Interestingly, they used a CRISPR/Cas approach to dissect the B cell receptor (BCR) signaling and its role in metabolism: although they did not directly focus on mTOR, interfering with PI3K signaling led to lower metabolic rates, both in term of glycolysis and OXPHOS [101]. This demonstrated how central the BCR/PI3K/Akt/mTOR axis is in regulating the bioenergetics status of these cells [101].

Despite the general agreement on their metabolism, there is however an unclear relationship between CLL cells and the stromal microenvironment: while there is evidence that the interaction with stromal cells induces a switch to glycolysis through a mechanism involving Notch and c-Myc [102], others observed an exacerbation of the respiratory rates, indicating an increase in OXPHOS after stromal contact [103]. 

mTOR role in the regulation of CLL cell metabolism has been studied especially in relation to the different response to therapeutic agents. Investigating the resistance to dasatinib, a second generation TKI, Marignac Martinez et al. reported a different regulation of the PI3K/Akt/mTOR pathway between sensitive and resistant primary cells: the first ones were characterized by a higher dependence on OXPHOS, a downregulation of PTEN and an upregulation of TCL1 [104]. In addition, both metformin, a respiratory chain complex I inhibitor, and rapamycin similarly synergized with dasatinib in inducing apoptosis in the sensitive subset [104]. Sharma et al. focused on the metabolic response to chemotherapeutic agent fludarabine: CLL cells, namely MEC-1 and 2 cell lines and primary samples showed an entirely similar profile to fludarabine resistant cells, a profound mTOR activation that caused an overall increase in glycolysis and OXPHOS rates, combined with an upregulation of purine biosynthesis [105]. An interesting observation was made by Siska et al.: chronic and acute B leukemia cells can induce metabolic changes in T lymphocytes by reducing their signaling through mTOR, thus slowing down their GLUT1-mediated glucose import and glycolytic rates, ultimately impairing their anti-leukemic action [106].

### 3.5. Multiple Myeloma

Multiple Myeloma (MM) plasma cells display a dependence on aerobic glycolysis for survival: they are characterized by an upregulation of GLUT family glucose transporter (namely GLUT4, 8 and 11) [107], a high expression of lactate dehydrogenase [108,109], and a carbon flux from glucose to lactate, with glutamine to replenish the TCA cycle [110]. The overexpression of GLUT transporter can be targeted by ritonavir, an antiretroviral protease inhibitor active against HIV [111]. The exposure to this agent can induce a downregulation of glycolysis and the concomitant dephosphorylation of mTOR, potentiated by the action of metformin [112]. Interestingly, metformin alone has been reported to deactivate mTOR signaling in MM cells, through the stimulation of AMPK [113]. 

Accordingly, interfering with the PI3K/Akt/mTOR pathway have a direct impact on glycolytic rates, as the exposure to BEZ235 (PI3K/mTOR dual inhibitor) impairs glycolysis, thus counteracting its upregulation by low concentration of topoisomerase inhibitors, such as doxorubicin, etoposide, and topotecan [114]. The mechanism of action of BEZ235 on glycolysis involves the downregulation of hexokinase II, the already mentioned glycolytic enzyme that is deeply involved in the acquisition of the Warburg effect metabolic rearrangement [115], which results overexpressed in myeloma cells [116]. As it occurs in AML and CLL, the microenvironment participates in the acquisition of metabolic changes, through the action of specific adhesion proteins. In MM, Reelin, a glycoprotein found on extracellular matrix, whose action is linked to cell proliferation and migration during development and in cancer [117], has been shown to stimulate mTOR signaling, which in turn increases glycolysis through the HIF1α upregulation [118]. mTOR signaling can also be inactivated in MM by targeting metabolic pathways that have been less studied, even though they are crucial in the energetic cellular balance, such as nicotinamide adenine dinucleotide (NAD) biosynthesis. NAD is in fact a cofactor that participates in a great variety of chemical reaction, acting as an electron shuttle [119]. Cea et al. reported that, inhibiting this rate-limiting enzyme involved in its formation, a reduction in the PI3K/Akt/mTOR activity can be observed, probably due to a deficit in cellular nutrient availability induced by the lack of accessible NAD [120].

### 3.6. Lymphomas

Lymphomas are subdivided in Hodgkin (HD) and non-Hodgkin (NHL). From the bioenergetic perspective, this categorization reflects a difference in their metabolism, as HD are associated with OXPHOS [121], while the NHL subtype are more prone to glycolysis [122], with some relevant exceptions. In Diffuse Large B Cell Lymphoma (DLBCL), belonging to the NHL class, a subset of patients’ cells shows in fact a transcriptional profile enriched in mRNAs participating in mitochondrial energy production, such as OXPHOS and electron transport chain machinery [123]. This observation has been confirmed at both proteomic and metabolic levels, with glucose- and fatty acid-derived carbon converging in the TCA cycle to generate the great proportion of cellular energy [124]. In general, evidences state that mTOR directly impacts on lymphoma glycolysis, as multiple works show that its targeted inhibition reduces the glycolytic phenotype. For example, primary effusion lymphoma (PEL), follicular and Burkitt Lymphomas have all been associated to high aerobic glycolysis and fatty acid synthesis, when compared to normal B cells [125]. This phenotype appears to be driven by the PI3K/Akt/mTOR module, since PI3K inhibition with LY294002 potently reduces both glycolysis and fatty acid synthesis (FAS) [125]. Interestingly, in normal B cells this inhibition causes the decrease of glycolysis, but not that of FAS, suggesting a different FAS regulation by the PI3K/Akt/mTOR axis between normal and pathologic cells [125]. Mediani et al. extended this study, confirming the highly glycolytic phenotype of PEL cells and its inhibition using the PI3K/Akt/mTOR inhibitors [126]. Moreover, they observed a switch in cell metabolism towards an oxidative phenotype when those cells are simultaneously exposed to a glycolysis inhibitor (2DG) and a dual PI3K/mTOR inhibitor (PF-04691502) [126]. This shift is, however, not sufficient to protect cells from the apoptosis synergistically induced by the two targeted agents [126]. Glycolysis inhibition through mTOR targeting has also been observed in mantle cell lymphoma (MCL). In fact, everolimus downregulated glucose transporters, glycolytic enzymes, and lactate dehydrogenase, thus inducing a decrease in lactate production [127], while the dual mTORC1/2 inhibitor AZD-2014 caused the activation of AMPK and the downregulation of glycolysis-related proteins [128]. Despite the already mentioned metabolic difference with the other NHL, in DBLCL interfering with the glucose catabolism has been reported to generate the same results, the mTOR inactivation, that in turn causes the downregulation of the pro-survival factor Mcl-1 [129]. However, a recent work by Chiche et al. subverted this picture, showing that mTORC1 is instead involved in the acquisition of the oxidative metabolism [130]. They observed that DLBCL can be subdivided into two categories, according to the expression levels of glyceraldehyde 3-phosphate dehydrogenase (GAPDH), an enzyme directly involved in the glycolytic pathway [130]. The high-expressing GAPDH cells were accordingly characterized by a glycolytic phenotype, while those cells that expressed GAPDH at low levels showed an OXPHOS preference, coupled with mTORC1 hyperactivation, fueled by glutamine [130]. Exposure to rapamycin impaired mitochondrial respiration while increasing glycolysis-derived ATP [130]. Interestingly, GAPDH overexpression inactivated mTORC1, thus, suggesting that this enzyme is implicated in the regulation of mTOR, at least in the DLBCL setting [118]. Table 3 summarizes the metabolic changes induced by different inhibitors.

## 4. Summary and Concluding Remarks

**Figure 2:**
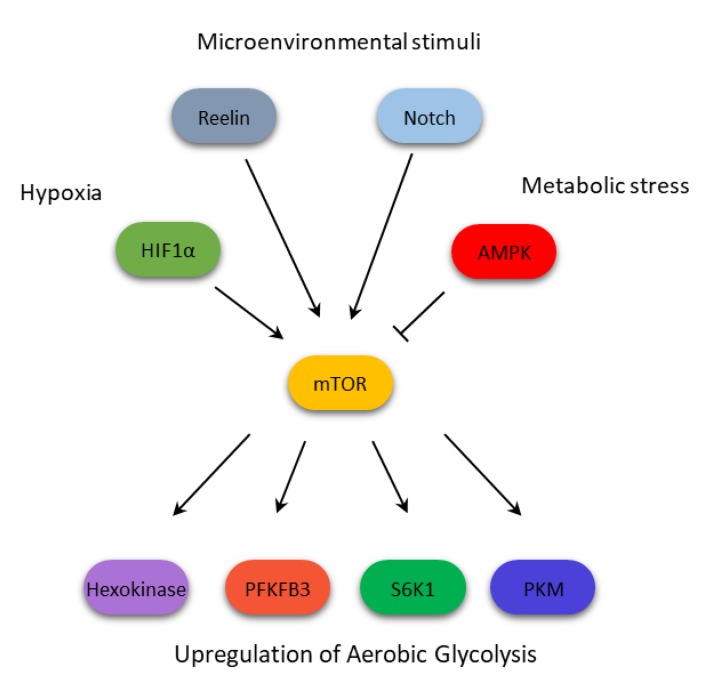
mTOR integrates multiple signals to confer a glycolytic phenotype on cells from hematologic malignancies.

As shown above, in hematologic malignancies the mTOR activation, working in opposition with AMPK but in concert with other oncogenes such as Bcr-Abl, or metabolic modulators like HIF1α, contributes to confer the glycolytic phenotype by directly and indirectly regulating key glycolytic enzyme activity (Figure 2). This metabolic activity has been linked to the acquisition of resistance to therapeutic agents. In our opinion, it would be important to predict and investigate the use of compounds active in reprogramming aberrant metabolic pathways, adopted in combination with standard treatments, for reducing the onset and impairing the resistance mechanisms developed by resistant cells. However, we are only beginning to understand the intricacy of the multiple regulation layers that contribute to the mTOR-mediated metabolic reprogramming. Additional studies are surely needed to shed light upon a mechanism that might constitute a major target to improve the current therapeutic arsenal of this group of hematologic malignancies.

## Figures and Tables

**Table 1:** Metabolic modulators in AML.

Compound	Target	Effect (Ref)
rapamycin	mTORC1	Decreased Glucose uptake (59);
		Shift from Glycolysis to OXPHOS (61);
		Decreased glycolysis through PFKB3 downregulation (65)
BKM-120	PI3K	Decreased Glycolysis and OXPHOS
2DG	Hexokinase	mTOR inactivation (62);
		mTOR activation (63)
L-asparaginases	Asparagine and glutamine degradation	mTOR inactivation (70)

**Table 2:** Targeting mTOR and metabolism in B- and T-ALL.

Type	Compound	Target	Effect (Ref)
B-ALL	everolimus	mTORC1	Decreased glycolysis and lactate generation (94)
	rapamycin	mTORC1	Decrease of glycolysis and increase of OXPHOS, reversion of thiaminase effects (96)
T-ALL	6-mercaptopurine	mTOR through AMPK activation	Decreased glucose and glutamine consumption (97)

**Table 3:** Targeting mTOR and metabolism in Lymphomas.

Type	Compound	Target	Effect (Ref)
PEL	LY294002	PI3K	Decreased glycolysis and FAS (125)
	PF04691502	PI3K/mTOR	Reduction of lactate production (126)
	Akti 1/2	Akt	Reduction of lactate production (126)
MCL	Everolimus	mTORC1	Reduction of lactate production (127)
	AZD-2014	mTORC1/2	Downregulation of glycolytic enzymes (128)
DBLCL	2DG	hexokinase	Inactivation of Akt/mTOR and decreased expression of Mcl-1 (129)
	rapamycin	mTORC1	Reduction of OXPHOS and increase of glycolytic ATP in the oxidative DBLCL subset (130)
